# Angekündigter assistierter Suizid in der Schweiz – Ein Fallbericht

**DOI:** 10.1007/s40211-021-00398-6

**Published:** 2021-09-27

**Authors:** Benjamin Vyssoki, Michaela Stich, Elisabeth Eder-Pissarek, Ingrid Jez, Stefan Dobias, Annemarie Unger, Alexander Kautzky, Georg Psota

**Affiliations:** 1 PSD Wien, Modecenterstr. 14/A2/2 OG, 1030 Wien, Österreich; 2 Klinische Abteilung für Sozialpsychiatrie, Univ. Klinik für Psychiatrie u. Psychotherapie, Währinger Str. 18–20, 1090 Wien, Österreich; 3 Praxis Dr. M. Stich, Am Schöpfwerk 31/6/R1, 1120 Wien, Österreich

**Keywords:** Assistierter Suizid, Euthanasie, Ethik, Persönlichkeitsstörungen, Assisted suicide, Euthanasia, Ethics, Personality disorders

## Abstract

Aktive Sterbehilfe ist aktuell in Österreich noch gesetzlich verboten. Aufgrund einer Entscheidung des Verfassungsgerichtshofs wäre assistierter Suizid ab dem Jahr 2022 gänzlich legal. Eine gesetzliche Regelung, die Grenzen zwischen legalen Formen einer Assistenz sowie deren Voraussetzungen und weiterhin unerlaubten Assistenzleistungen zieht, ist derzeit noch nicht in Sicht. Ein Suizid, der auf Verlangen des Suizidenten von fremder Hand herbeigeführt wird, bleibt jedenfalls weiterhin strafbar; dies auch dann, wenn der Suizident physisch nicht mehr in der Lage ist, bei seiner Tötung mitzuwirken. In mehreren europäischen Ländern wurde aktive Sterbehilfe bereits legalisiert und in manchen Ländern, wie zum Beispiel der Schweiz, kann assistierter Suizid auch ohne lebenszeitlimitierende Grunderkrankung, bei unerträglichem Leid und insuffizient vorhandenen weitere Behandlungsmöglichkeiten in Anspruch genommen werden. In diesem Fallbericht wird die Kasuistik einer Patientin geschildert welche, als Grunderkrankung an einer Persönlichkeitsstörung leidend, einen assistierten Suizid in der Schweiz geplant hatte. Es werden die ethischen und juristischen Hintergründe dieses Fallberichts diskutiert.

Das zur Verfügung stellen assistierter Suizidmöglichkeiten birgt die große Gefahr, dass Menschen mit psychischer Erkrankung, insbesondere bei schweren depressiven Episoden, Behandlungsangebote ablehnen und anstelle Suizid durch einen professionellen Sterbehilfeanbieter wählen könnten. Insbesondere gewarnt werden muss vor der Tatsache, dass Menschen in aktuell schwer depressivem Zustandsbild aufgrund dessen zumeist unter eingeschränkter Entscheidungsfähigkeit leiden und nur bedingt über die Möglichkeit der freien Willensentscheidung verfügen.

## Abstract (en)

Assisted suicide is currently still prohibited in Austria. However, following a finding of the Austrian Constitutional Court it would be completely legal from 2022. A statutory regulation defining the limits between legal ways of assistance and the conditions therefor on the one hand and, on the other hand, assistive actions that shall remain prohibited is currently not in sight. However, a suicidal action performed upon the request of the person willing to die solely by someone else will remain illegal; this also applies if the person willing to commit suicide is physically unable to participate in her or his killing. In several European countries euthanasia and/or assisted suicide has already been legalized and in some countries, Switzerland for example, assisted suicide is a legal option even for patients without suffering from a life-limiting disease, unbearable suffering and insufficient treatment options are the only criteria. In this case report the clinical case of an Austrian patient will be presented, suffering from a personality disorder, who planed an assisted suicide in Switzerland. Ethic and legal backgrounds are discussed.

The possibility of assisted suicide bears the danger that people suffering from psychiatric disorders, especially current major depressive episode, could refuse treatment options and choose suicide trough a commercial provider of assisted suicide.

In particular it must be considered that currently severely depressed people usually suffer from limited insight and judgement as well as from limited freedom of choice.

Die Behandlungshoheit des Einzelnen umfasst neben der Ablehnung von lebenserhaltenden Maßnahmen insbesondere das Recht auf ein menschenwürdiges Sterben sowie das Recht, Hilfe eines Dritten in Anspruch zu nehmen [1]. Dieses vom österreichischen Verfassungsgerichtshof mit 11.12.2020 gefällte Erkenntnis, welches mit Ablauf des 31.12.2021 die Rechtslage ändern wird, schafft erstmals die rechtliche Grundlage Beihilfe zum Suizid in Österreich straffrei in Anspruch zu nehmen (wobei abzuwarten bleibt, auf welche Weise der Gesetzgeber die neue Rechtslage im Detail gestalten wird). Nach derzeitiger Rechtslage ist in Österreich (gemäß § 78 Strafgesetzbuch [StGB]) jede Form der Hilfeleistung zum Suizid mit einer Freiheitsstrafe von sechs Monaten bis zu fünf Jahren bedroht. Dies steht im Gegensatz zur Rechtslage in mehreren europäischen Ländern, in welchen bereits eine Legalisierung der aktiven Sterbehilfe und/oder assistierten Suizids erfolgte. Diese Länder sind die Niederlande, Belgien, Luxemburg und die Schweiz, wobei außerhalb Europas auch in Kolumbien, Kanada sowie mehreren Bundesstaaten der USA und einem Bundestaat in Australien Beihilfe zum Suizid straffrei ist [2]. In der Schweiz, den Niederlanden und Belgien kann assistierter Suizid auch bei einer nicht lebenszeitverkürzenden Erkrankung in Anspruch genommen werden. Ausreichend sind hierfür unerträgliches Leid und insuffizient vorhandene weitere Behandlungsmöglichkeiten [2]. Daher ist es auch für Menschen, die „nur“ an psychischen Erkrankungen leiden, nach diesen Kriterien möglich, einen Antrag auf assistierten Suizid zu stellen. Des Weiteren ist es auch für Menschen aus Österreich möglich, vor Ort, z. B. in der Schweiz, nach diesen Kriterien legal assistierten Suizid in Anspruch zu nehmen. So wurden in den Arbeiten von Fischer et al. [3] und Gauthier et al. [4] explizit auch die Herkunftsländer der Menschen, welche sich in der Schweiz mit Hilfe eines Dritten suizidierten, untersucht und Menschen aus Österreich aufgelistet: bei Fischer fanden sich im Zeitraum 2001–2004 sieben Österreicherinnen und Österreicher, bei Gauthier 14 im Zeitraum 2008–2012.

Im vorliegenden Fall berichten die AutorInnen über eine Patientin, welche einen assistierten Suizid in der Schweiz geplant hatte, und diskutieren weiterführend die ethischen und juristischen Hintergründe zu diesem Thema. In der Schweiz wird Beihilfe zum Suizid seit 1985 angeboten, wobei aktuell über fünf Organisationen hier tätig sind – mit heterogenen Kriterien, unter welchen Bedingungen im Hinblick auf Alter und Grunderkrankung Menschen ihr Angebot in Anspruch nehmen dürfen; die Kosten dafür schwanken und liegen zwischen freiwilliger Spende bis hin zu mehreren tausend Euro/Schweizer Franken [5].

Zur Patientin: Frau MM nahm im Alter von Mitte 30 Jahren erstmals psychiatrische Behandlung im niedergelassenen Bereich in Anspruch. Sie berichtete über eine depressive Stimmungslage, orthorektisches Essverhalten, Kleptomanie sowie über psychische Belastung aufgrund einer unzufriedenstellenden Beziehung. Auffallend war auch ein histrionisches Verhalten.

In den folgenden Jahren kam die Patientin nur in unregelmäßigen Abständen in die Praxis; zur Behandlung der depressiven Störung erfolgte eine medikamentöse antidepressive Therapie, worunter es zu einer weitgehenden Remission kam. Im weiteren Verlauf waren jedoch eine Aggravierung des histrionischen Verhaltens sowie der Kleptomanie beobachtbar. Über einen Zeitraum von sechs Jahren wurden mehrere Psychotherapien begonnen und wieder abgebrochen. Die Patientin berichtete über wechselnde Beziehungen und berufliche Tätigkeiten.

Als die Patientin fast 40 Jahre alt war, waren aufgrund mehrerer Diebstahlversuche Anzeigen erstattet worden und es kam zu einem Gerichtsverfahren. Dies hatte eine schwere depressive Symptomatik zu Folge, wobei die Patientin zusätzlich die antidepressive Medikation absetzte. Einen weiteren Psychotherapieversuch verweigerte sie wiederholt mit der Begründung, die Therapie hätte früher stattfinden müssen, jetzt könne man nicht mehr helfen. Auch eine stationäre Psychotherapie oder Spitalsbehandlung lehnte Frau MM ab.

Die Patientin zog sich zunehmend zurück und verließ schließlich die Wohnung gar nicht mehr. Trotzdem fanden weiterhin wöchentliche psychiatrische Telefonkontakte statt, wobei die Patientin jedoch jede intensivere Form der Behandlung ablehnte. Es kam zu einem zunehmenden Selbstfürsorgedefizit und Frau MM verließ über fast ein Jahr die Wohnung nicht. Frau MM wurde mehrere Monate täglich von ihren Eltern mit Essen versorgt. Es wurde zweimal eine amtsärztliche Einweisung auf die zuständige psychiatrische Regionalabteilung erwirkt, die Patientin jedoch beide Male nicht stationär aufgenommen.

Nach etwa einem Jahr, in welchem die Patientin die Wohnung nicht verlassen hatte, berichtete Frau MM, sich bei einem Institut für Sterbehilfe in der Schweiz beworben zu haben und als „Kandidatin“ angenommen worden zu sein. Es sei der Patientin auch eine Preisreduktion von mehreren tausend Euro in Aussicht gestellt worden, wenn sie sich schnell für einen Termin entscheide. Daraufhin leistete die Patientin eine Anzahlung und erhielt einen Termin.

Es fand, nach Einverständnis der Patientin, ein Telefonat der behandelnden Fachärztin mit dem schweizer Gutachter (ein Facharzt für Kinder- und Jugendpsychiatrie) statt, der die Patientin via Videokonferenz begutachtet hatte. Bei diesem Gespräch wurde der Psychiaterin mitgeteilt, dass die Patientin nach Ansicht des Gutachters alle Kriterien erfüllen würde, Sterbehilfe in diesem Institut in Anspruch nehmen zu können. Eine schwere Persönlichkeitsstörung sei aufgrund der „schlechten Prognose“ ein ausreichender Grund für Sterbehilfe.

Zeitgleich mit der, von der Patientin durchgeführten, Anmeldung zum assistierten Suizid, suchten die Angehörigen der Patientin beim Psychosozialen Dienst der Stadt Wien (PSD-Wien) um Hilfe und Intervention an. Darauf erfolgte eine Kontaktaufnahme des PSD-Wien mit der behandelnden Fachärztin zwecks Planung und Akkordieren des weiteren Procederes, insbesondere Klärung der rechtlichen Rahmenbedingungen durch die Rechtsabteilung des PSD-Wien und Erörterung einer Einweisung nach dem Unterbringungsgesetz (UbG): Ein Suizidversuch für sich genommen ist kein zwingendes Symptom einer psychischen Krankheit; dasselbe gilt für ernstzunehmende Selbsttötungsabsichten. Es muss daher im Einzelfall geprüft werden, ob die psychische Krankheit für den Todeswunsch (mit)kausal ist, deshalb die Entscheidungsfähigkeit zu einem solchen Entschluss nicht vollumfänglich gegeben ist, oder, ob der Wunsch auf freier Entscheidung bei erhaltener Entscheidungsfähigkeit beruht. Im ersten Fall ist die Unterbringung (bei fehlenden gelinderen Mitteln) zulässig, sofern die Wahrscheinlichkeit eines weiteren Suizidversuchs, und damit eine ernstliche Selbstgefährdung, vorliegt. Im vorliegenden Fall wurde der Todeswunsch von ärztlicher Seite auf die psychische Erkrankung zurückgeführt.

Die von der erkrankten Person ausgehende Gefährdung muss für eine Unterbringung „ernstlich und erheblich“ sein. Damit ist nicht nur ein hohes Maß an Wahrscheinlichkeit des Schadenseintritts angesprochen, sondern auch eine besondere Schwere der drohenden Schädigung. Die Gefahr ist umso „ernstlicher und erheblicher“, je größer die Wahrscheinlichkeit oder die Höhe des Schadens ist. Im vorliegenden Fall war eine besondere Schwere der Schädigung (Tod der Patientin) zweifelsfrei gegeben und die Wahrscheinlichkeit ihres Eintritts als hoch zu bewerten, da die Patientin bereits einschlägige Maßnahmen ergriffen hatte (Anzahlung, Gutachten) und der assistierte Suizid in wenigen Tagen stattfinden sollte. Diesbezüglich wurde in der letzten großen Novellierung des UbG betont, dass ein Schadenseintritt nicht unmittelbar bevorstehen, sondern „innerhalb eines absehbaren Zeitraums (von einigen Wochen bis wenigen Monaten)“ zu erwarten sein muss [6].

Von einer sogenannten „Garantenstellung“, also einer besonderen Rechtspflicht zu einem positiven Handeln, um den Eintritt eines Schadens abzuwenden, sind ÄrztInnen nur dann entbunden, wenn eine ausreichend entscheidungsfähige PatientIn eine lebenserhaltende oder -verlängernde ärztliche Behandlung ablehnt. Die dafür notwendige Entscheidungsfähigkeit war im vorliegenden Fall nicht gegeben und daher eine Unterbringung zu veranlassen.

Es erfolgte eine amtsärztliche Zuweisung und in weiterer Folge eine psychiatrisch stationäre Aufnahme sowie Unterbringung ohne Verlangen an der Universitätsklinik für Psychiatrie und Psychotherapie Wien. Bei der Unterbringungsverhandlung wurde die Zulässigkeit der Unterbringung durch das Gericht bestätigt.

Die Festlegung der rechtlichen Rahmenbedingungen, unter welchen Umständen Beihilfe zum Suizid in Anspruch genommen werden wird können, ist in Österreich noch nicht erfolgt. Von besonders hoher Relevanz in diesem Zusammenhang ist, inwiefern der Gesetzgeber festschreiben wird, dass assistierter Suizid nur bei stark verkürzter Lebenserwartung und „unerträglichem Leid“ in Anspruch genommen werden darf, oder ob „unerträgliches Leid“ ohne Behandlungsmöglichkeit als Kriterium ausreichend wäre, wie dies beispielsweise bereits in der Schweiz oder den Niederlanden der Fall ist. Falls auch ohne lebenszeitverkürzende Erkrankung Beihilfe zum Suizid in Anspruch genommen werden könnte, würde hierdurch die Gruppe der Personen, welche sich rechtskonform beim Suizid assistieren lassen könnten, massiv erweitert.

Besonders problematisch wäre dies für Menschen mit psychischen Erkrankungen, da es, insbesondere bei affektiven Störungen und bei Persönlichkeitsstörungen, zu häufigem, aber auch langanhaltenden, Wunsch nach Suizid kommen kann – so wie auch in der geschilderten Kasuistik. Dies ist von besonderer gesundheits- und sozialpolitischer Relevanz, da affektive Störungen und Persönlichkeitsstörungen zu den häufigsten psychischen Erkrankungen zählen [7].

Die Befürchtung wäre des Weiteren, dass Menschen in einer akuten depressiven Episode eine Behandlung derselben ablehnen und anstelle Suizid begehen wollen würden – aufgrund der subjektiven Ausweglosigkeit, bei jedoch gleichzeitig mit der Depression häufig assoziierten eingeschränkten Entscheidungsfähigkeit. Ähnliches ist auch bei Menschen mit schweren Persönlichkeitsstörungen zu befürchten: wo erfolgreiche Behandlungsprozesse nicht selten Jahre benötigen, wo in Episoden der Krise assistierter Suizid als rechtlich konformer und gesellschaftlich akzeptierter vermeintlicher Ausweg erscheinen könnte.

Bereits gezeigt wurde, dass in den Ländern mit dem Angebot zu Sterbehilfe bei psychischer Grunderkrankung eine Steigerung in der Inanspruchnahme während des untersuchten Zeitraums beobachtet werden konnte [8]. In einer rezenten Literaturübersichtsarbeit aus dem Jahr 2021 haben Calati et al. sich diesem Thema gewidmet [2]. In dieser Arbeit erforschen die AutorInnen, ob es Daten gibt, mit welcher Häufigkeit Menschen mit psychiatrischer Erkrankung assistieren Suizid in jenen Ländern in Anspruch genommen haben, wo es rechtlich legalisiert wurde, und inwiefern diese klinisch beschrieben werden können. Calati et al. kommen zum Schluss, dass es große Ähnlichkeit gibt zwischen den Menschen, welche Beihilfe zum Suizid in Anspruch genommen haben, mit den sog. „traditionellen“ psychiatrischen „HochrisikosuizidpatientInnen“. Ihre Ergebnisse interpretieren die AutorInnen dahingehend, dass die Möglichkeit zu assistiertem Suizid insbesondere bei Frauen zu vermehrten Suiziden führen würden, welche ansonsten verhinderbar gewesen wären. Dies aufgrund des zur Verfügung Stellens einer 100 % letalen Suizidmethode bei aufgrund einer psychiatrischen Grunderkrankung suizidal eingeengten Menschen. Calati et al. betonen, dass sich in den untersuchten PatientInnendaten auch eine hohe Rate von Personen fand, welche nach angenommener Anmeldung zum assistierten Suizid ihre Entscheidung revidierten und sich gegen ihren Suizid entschieden, fast 40 %! Evenblij et al. (2019) untersuchte jene Menschen, deren Ansuchen nach aktiver Sterbehilfe verweigert wurde; hier zeigte sich, dass 69 % der Menschen weiter am Leben blieben, 16 % starben durch Suizid im Nachbeobachtungszeitrum. Diese Zahlen verdeutlichen die Wandelbarkeit in der Entscheidung zur Inanspruchnahme von Sterbehilfe bei Menschen mit psychischer Erkrankung [9].

Falls es zu einer Legalisierung der Sterbehilfe in Österreich kommen sollte, welche, wie in den Benelux-Ländern auch Menschen mit rein psychischen Erkrankungen umfasst, ist auf die unbedingte Notwendigkeit einer unabhängigen interdisziplinären Kommission hinzuweisen [2]. Diese Kommission würde besagte Anträge evaluieren und müsste unbedingt eine fachärztliche psychiatrische Expertise beinhalten, welcher auch die Kompetenz der Letztentscheidung zukommt. Zudem muss auch eine Begutachtung des Längsschnittes erfolgen, da dies insbesondere auch bei Menschen mit Persönlichkeitsstörungen für eine exakte Diagnostik notwendig ist [10].

Am Wichtigsten erscheint den AutorInnen dieses Artikels aber zukünftig noch mehr Ressourcen in die adäquate Prävention, Diagnostik und multimodale Behandlung von Menschen mit psychischen Erkrankungen zu investieren, um schweres psychisches Leid zu behandeln, zu mildern und hiermit zu erreichen, dass der Wunsch nach Sterbehilfe überhaupt erst gar nicht entsteht. In diesem Zusammenhang wichtig ist auch der Ausbau und die Weiterentwicklung aufsuchender psychiatrischer Krisen- und Notfallversorgung, ambulanter Palliativpsychiatrie sowie von suizidpräventiven Maßnahmen. Insbesondere im Bereich der Kinder- und Jugendpsychiatrie, der Transitionspsychiatrie, sowie in der Behandlung von akuten Traumata sollten bestehende Behandlungsnetzwerke erweitert und neue aufgebaut werden, um hier einerseits umfassend bei Heranwachsenden gezielt professionell therapeutisch intervenieren zu können und andererseits bei akuten Traumata die Weiterentwicklung zu chronischen Traumafolgestörungen verhindern zu können.

Letztlich scheint es den AutorInnen von besonderem Belang, dass gerade Menschen mit schweren psychischen Erkrankungen in bestimmten kritischen Phasen nicht über die Möglichkeit der freien Willensentscheidung verfügen. Es liegt an uns als Gesellschaft sie diesbezüglich vor Schaden zu bewahren und uns nicht ihrer – mit dem Argument „sie haben es ja so gewollt“ – zu entledigen. Kein anderes medizinisches Fach ist – allein schon aus seiner Historie heraus – mehr als die Psychiatrie gefordert dergleichen Entwicklungen kritisch zu hinterfragen.
